# Application of Optimization Algorithms in Clusters

**DOI:** 10.3389/fchem.2021.637286

**Published:** 2021-03-12

**Authors:** Ruby Srivastava

**Affiliations:** Bioinformatics, CSIR-Centre for Cellular and Molecular Biology, Hyderabad, India

**Keywords:** global optimization, potential energy landscape, clusters, empirical potentials, homotops

## Abstract

The structural characterization of clusters or nanoparticles is essential to rationalize their size and composition-dependent properties. As experiments alone could not provide complete picture of cluster structures, so independent theoretical investigations are needed to find out a detail description of the geometric arrangement and corresponding properties of the clusters. The potential energy surfaces (PES) are explored to find several minima with an ultimate goal of locating the global minima (GM) for the clusters. Optimization algorithms, such as genetic algorithm (GA), basin hopping method and its variants, self-consistent basin-to-deformed-basin mapping, heuristic algorithm combined with the surface and interior operators (HA-SIO), fast annealing evolutionary algorithm (FAEA), random tunneling algorithm (RTA), and dynamic lattice searching (DLS) have been developed to solve the geometrical isomers in pure elemental clusters. Various model or empirical potentials (EPs) as Lennard–Jones (LJ), Born–Mayer, Gupta, Sutton–Chen, and Murrell–Mottram potentials are used to describe the bonding in different type of clusters. Due to existence of a large number of homotops in nanoalloys, genetic algorithm, basin-hopping algorithm, modified adaptive immune optimization algorithm (AIOA), evolutionary algorithm (EA), kick method and Knowledge Led Master Code (KLMC) are also used. In this review the optimization algorithms, computational techniques and accuracy of results obtained by using these mechanisms for different types of clusters will be discussed.

## Introduction

**FIGURE 1:**
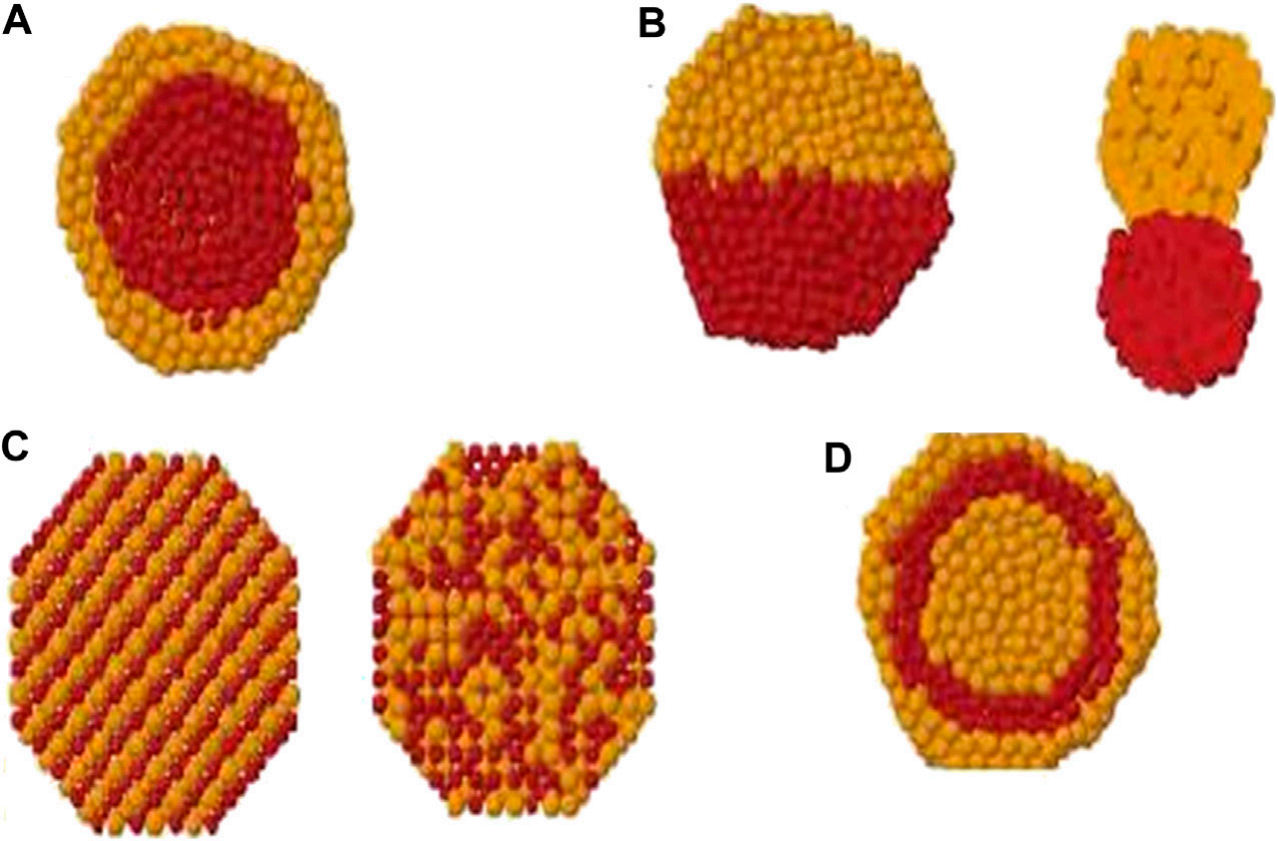
Cross section view of **(A)** core-shell, **(B)** subcluster segregated, **(C)** mixed and **(D)** multishell patterns. (Ismail, 2012).

Nanoclusters are considered as a collection of ∼10 to 10^6^ atoms or molecules within a nanometre size range (Lesley and Johnston, 2000; Johnston, 2002) such as fullerenes, metal clusters, molecular clusters and ionic clusters (Jellinek, 1999; Baletto and Ferrando, 2005). Nanometre-size clusters are both crystalline (face-centred cubic (fcc), octahedra or truncated octahedral (TO)) and noncrystalline (icosahedra, decahedra, polytetrahedra and polyicosahedra) structures. The small size nanoclusters exist in noncrystalline shapes. The noble and transition metals are dominated with icosahedra and Marks truncated decahedra structures (Martin, 1996). These structures are not favourable for large clusters due to the strain arising from their noncrystalline packing (Baletto and Ferrando, 2005). However the strain can be released by placing a smaller atom in the core of the nanoalloy (Rossi et al., 2004) as the strain is proportional to the cluster volume. The quantized electronic energy levels of clusters give rise to atomic-like character (Halperin, 1986; Ralph et al., 1995) and this phenomenon is used to enhance the optical and electrical properties of some clusters (Heath, 1995; Papavassiliou, 1979). Advancement in modern research occur with nanoalloy composition (Ferrando et al., 2008; Oderji and Ding, 2011; Liu et al., 2005) and chemical ordering patterns (Scott et al., 2004; Chen et al., 2005; Knudsen et al., 2007; Maksimuk et al., 2007; Ye and Crooks, 2007; Teng and Yang, 2003) in addition to the size, atomic order and structure. Chemical ordering depends upon structure, size and composition, among others (Johnston, 2003). See Figure 1. The theoretical studies for clusters are far cheaper than the experimental trial-and-error approaches, and led to conclusion by following parameters; heat of formation, energies, structural mechanisms, transition states (TS) mechanisms, and molecular spectra analysis (Foresman and Frisch, 1996). Various groups have explored these properties for the generation and characterization of the atomic clusters (Cabaleiro-Lago et al., 2000; Marques and Pereira, 2010a; Marques and Pereira, 2013; Marques and Pereira, 2013, Marques et al., 2018; Bartolomei et al., 2015).

The PES is explored to locate the GM for the smaller clusters by different approaches. Electronic structure of these clusters can be defined by either *ab initio* Molecular Orbital (MO) or semi-empirical atomistic potentials (Wales and Scheraga, 1999; Johnston, 2002). GM structure is the most preferred structure though other metastable structures are also observed due to kinetic effects. A*b initio* method are feasible for the smaller systems (tens of atoms) as it is based on the laws of quantum mechanics, atomic, electronic properties and few other physical constants. In this method, the system is studied either by short single-point energy calculation or by geometrically relax the system for more stable configuration. The empirical or semi-empirical methods are used for the medium sized systems. As *ab initio* methods are computationally demanding so empirical methods are used as an alternative, but empirical methods are not found to be accurate to encounter hydrogen-bonding, chemical transitions or nitrated compounds (Akutsu et al., 1991; Cook, 2005). Further compared to pure elemental clusters, the existence of homotops complicates nanoalloys studies. The conformational stability of clusters is carried out by the global minima and transition states being stationary points with zero potential gradients. Local minima is obtained by the rise in potential energy for any infinitesimal displacement of internal coordinates, where there is a Hessian with positive (second derivative matrix) eigenvalue for the transition states.

GA is considered as a better and popular choice in clusters compared to Monte Carlo (MC) and Molecular Dynamics (MD) Simulated Annealing method. The other techniques are evolution strategies, differential evolution, genetic programming, evolutionary programming, gene expression programming, neuro-evolution, learning classifier systems. Xiao and Williams (Xiao and Williams, 1993) used the GA approach for the molecular clusters (benzene, naphthalene and anthracene) in 1990’s. Then Hartke (Hartke, 1993) reported the genetic algorithms for global optimization of molecular clusters. The binary encoded geometries and bitwise acting genetic operators on binary strings were reported by Xiao et al. (Xiao and Williams, 1993). Further these binary encoding and decoding were replaced by applying cartesian coordinates on GA approach (Zeiri, 1995). A significant contribution was made by Deaven and Ho (Deaven and Ho, 1995) in which gradient-driven local minimization was implemented for the cluster energy. Birmingham Cluster Genetic Algorithm (BCGA) in house GA was developed by Wales group for Morse clusters (Roberts et al., 2000), fullerenes (Johnston, 2003), ionic clusters (Roberts and Johnston, 2001), water clusters (Guimaraes et al., 2002), metal clusters (Lloyd et al., 2002) and bimetallic clusters (Bailey et al., 2003; Lordeiro et al., 2003). BHMC algorithm is based on the MC minimization or BH algorithm (Li and Scheraga, 1987) in which PES is simplified by the transformation of energy which results in a smoother landscape. So these methods are also known as hypersurface deformation (Stillinger and Weber, 1988). In 2005 Karaboga (Karaboga, 2005) proposed artificial bee colony (ABC) algorithm which was very efficient in locating global minima for long range potentials. The ABC algorithm was successfully applied to the atomic clusters (Zhang and Dolg, 2015) and rigid molecules with corresponding ABCluster software (Zhang and Dolg, 2016). Particle swarm optimization (Call et al., 2007), stochastic surface walking (Shang and Liu, 2013), kick method (Saunders, 1987; Bera et al., 2007; Addicoat and Metha, 2009; Zhai et al., 2015) and GIGA (Jäger et al., 2019) have shown good performance for various chemical systems.

**FIGURE 2:**
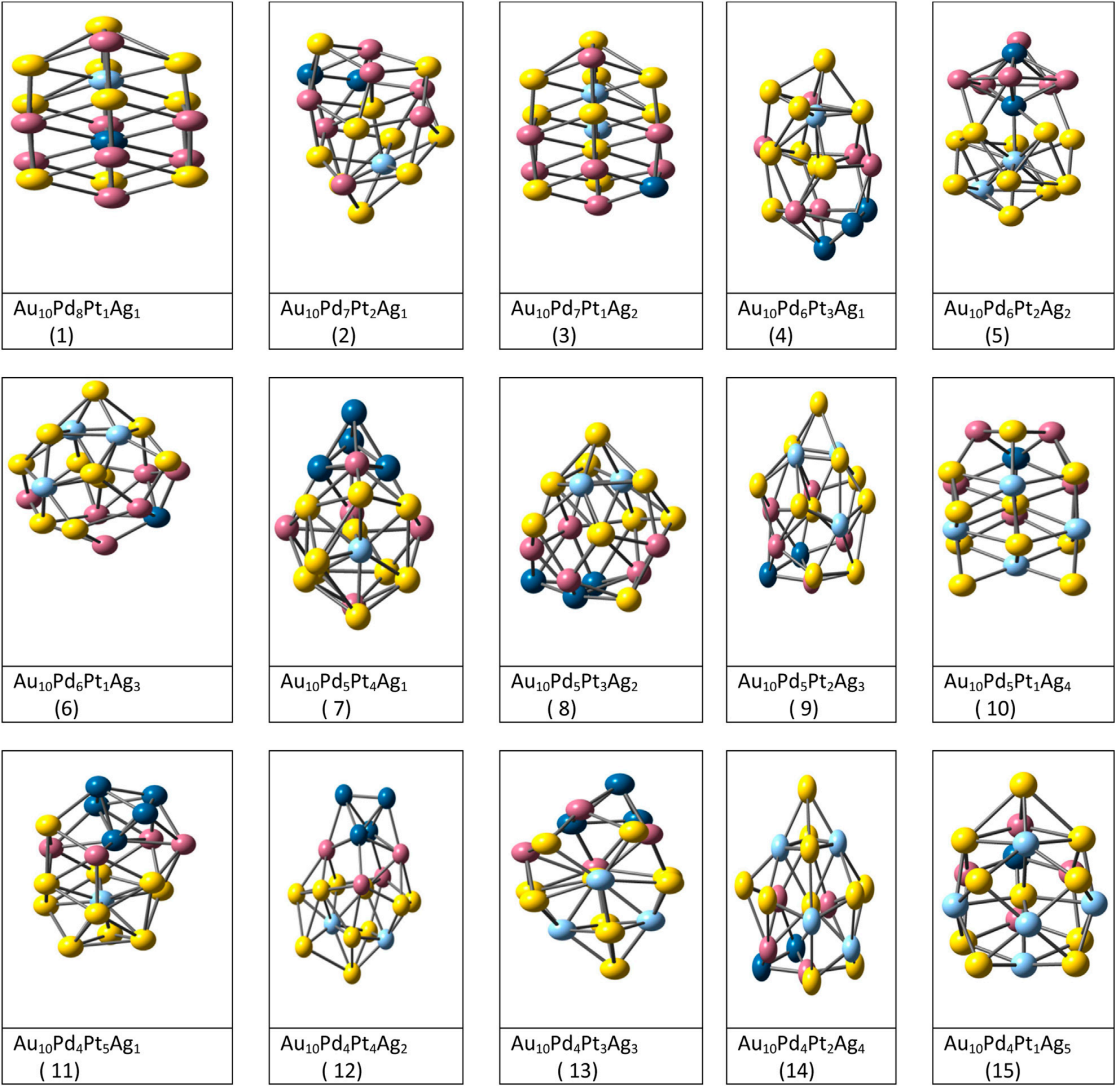
Optimized structures of Fifteen tetrametallic clusters complexes by DFT method. (Srivastava, 2017b).

Various model or empirical potentials (EPs) are used to describe the bonding in these clusters. It was observed that global minimum configurations show different symmetries for Sutton Chen potentials and Lennard-Jones potentials. The Sutton-Chen potential is a Finnis-Sinclair type potential with two terms providing the pair-wise repulsive and approximate many-body cohesive contributions separately. Gupta potential is a semi-empirical potential derived within the tight-binding second-moment approximation. It is highly recommended for metallic systems (Gupta, 1981) with inter-atomic interactions. This potential function is applied to describe homonuclear and heteronuclear interactions. The parameters used for this potential is given in Table 1 from the work carried out by Srivastava (Srivastava, 2017a) for (Au_m_-Ag_n_-Pd_o_-Pt_p_) (m = 10 and n + o + *p* = 10) tetrametallic clusters. A, ξ, p and q potential parameters are used to fit the experimental properties as cohesive energy, lattice parameters, elastic constants, among others), while r_0_ can either be average of the pure bulk distances or can be taken by specific ordered bulk alloy. See Figure 2.

**TABLE 1:** Gupta potential parameters used for the four (Pt, Pd, Ag, Au) coinage metals. (Srivastava, 2017b).

Compositions	A_*ij*_ (eV)	ξ_*ij*_ (eV)	*p*_*ij*_	q_*ij*_	r_ij_^(0)^ (Å)
Pt-Pt	0.2975	2.6950	10.612	4.004	2.7747
Pt-Pd	0.2300	2.2000	10.740	3.870	2.7600
Pd-Pd	0.1746	1.7180	10.867	3.742	2.7485
Ag-Ag	0.1028	1.1780	10.928	3.139	2.8885
Ag-Au	0.1490	1.4874	10.494	3.607	2.8864
Au-Au	0.2061	1.7900	10.229	4.036	2.8843
Au-Pt	0.2500	2.2000	10.420	4.020	2.8300
Ag-Pd	0.1607	1.5597	10.895	3.492	2.8230
Ag-Pt	0.1750	1.7900	10.730	3.590	2.8330
Au-Pd	0.2764	2.0820	10.569	3.913	2.8160

Due to high computational costs, a combined empirical-*ab initio* approach is used to carry out the unbiased searches at empirical level for global and local minima structures. These structures were optimized at *ab initio* level and GO technique was used at EP level to build a database of structural motifs. Global searches were performed with the BCGA and BHMC algorithms by employing Gupta and Gupta-derives potentials. Then the selected minima are locally optimized at the DFT level using various softwares as the Northwest Computational Chemistry (NWChem) (Aprà et al., 2020), Quantum ESPRESSO (QE) (Giannozzi et al., 2020), Gaussian 09 package (Frisch, 2009), VASP (Kresse and Furthmüller, 1996) and ADF (TeVelde et al., 2001; Baerends et al., 2003) etc.

OGOLEM (Hartke, 1993; Dieterich and Hartke, 2017), GMIN (Wales and Scheraga, 1999; Wales, 2010), BCGA (Johnston, 2003; Shayeghi et al., 2015), Gradient Embedded Genetic Algorithm or GEGA (Alexandrova and Boldyrev, 2005), Global Reaction Route Mapping (GRRM) (Ohno and Maeda, 2006; Ohno and Maeda, 2019), Evolutionary Algorithm for Molecular Clusters or EA_MOL (Llanio-Trujillo et al., 2011; Marques and Pereira, 2011), Automated Mechanisms and Kinetics (AutoMeKin) (Martínez-Núñez, 2015a; Martínez-Núñez, 2015b; Martínez-Núñez et al., 2020), ABCluster (Zhang and Dolg, 2015), Genetic Algorithm fitting (GAFit) (Rodríguez-Fernández et al., 2017; Rodríguez-Fernández et al., 2020), AUTOMATON (Yañez et al., 2019; Yañez et al., 2020) and NWPEsSe (Zhang et al., 2020) are some of the computational tools which have included many of these methods.

The energetic analysis of clusters is carried out by average binding energy (pure clusters), second difference in binding energy and excess (or mixing) energy for nanoalloys by both EP and DFT levels. The mixing effect is studied by various factors as size, cohesive energy, surface energy, electronegativity among many others. Radial distribution function (RDF), pair distribution function (PDF) and average nearest-neighbour distance (ANND) are also calculated for bonding analysis. Further chemical ordering and symmetry are studied for the cluster structure analysis. Compositional Mixing Degree is calculated to give emphasis to the mixed bonds (Srivastava, 2017a; Srivastava, 2018a).

In next section, we will discuss the use of these optimization algorithms in various types of clusters.

## Pure Metallic Clusters

Spherical shell model was used to determine the electronic structures of “magic numbers” in Na_n_ and K_n_ alkali-metal clusters (Brack, 1993; de Heer, 1993). Åkeby et al. (Åkeby et al., 1990) used the configuration interaction (CI) method with an effective core potential for small clusters (n ≤ 10). Full-potential muffin-tin orbitals (FP-LMTO) technique was used for small Cu_n_ clusters (Kabir et al., 2003). Tight-binding (TB) approach with quasi-empirical potential was used to study the molecular dynamics for nearly 1,300 atoms (D’Agostino, 1993). A minimal parameter TBMD method was used for transition metal (Ni_n_ and Fe_n_) clusters (Menon et al., 1994; Lathiotakis et al., 1996). Random search method has been adopted by Johnston group on (Al, Ca, Fe, Ni, Pd and Pt) bound 17–19 atoms clusters by Murrell–Mottram 2 + 3 body potentials. Results indicated similar structural patterns but different positions of elements for both Murrell–Mottram and Sutton–Chen potentials.

A previous studies indicated amorphous structures for 13 atoms (Au, Ag, Cu) clusters (Oviedo and Palmer, 2002) and large Au_n_ clusters (n = 38, 55, 75) (Michaelian et al., 1999; Li et al., 2000; Wilson and Johnston, 2000). *Ab initio* studies showed that most of the copper clusters adopt icosahedral structure for (10 ≤ n ≤ 55), derived from the 13 atom icosahedron; polyicosahedral (19, 23, and 26) atom; and icosahedron (55 atoms) clusters by adding or removing atoms (Moore, 2013). Small anionic gold clusters were studied by Häkkinen et al. (Häkkinen et al., 2002) with PBE (Perdew et al., 1996) functional in which Au_7_^−^ formed a planar structure, whereas both Cu_7_^−^ and Ag_7_^−^ form 3D structure. Furche et al. (Furche et al., 2002) found planar structures for Au_*n*_^−^ up to *n* = 15. DFT calculations find stable planar gold clusters (Jain, 2005), while *Fa et al.* (Fa et al., 2005) predicted 2D*→*3D transition between *n* = 13 and *n* = 15 for neutral gold clusters.

Large magic number Cu clusters tend to adopt close-packed structures such as icosahedron or cuboctahedron (Massobrio et al., 1998). Jackson used local-spin-density approximation for even-numbered Cu clusters, while other study (Winter et al., 1991) predicted jellium model like icosahedral geometrical closure effects for small copper clusters. Density functional theory (DFT) within generalized gradient approximation and BFGS algorithm on Cu_20_, Ag_20_, and Au_20_ clusters study also showed tetrahedral structures with T_d_ symmetry for Ag_20_ and Au_20_ clusters (Wang et al., 2003). Li et al. (Li et al., 2003) predicted tetrahedral structure with T_d_ symmetry for Au_20_; similar to alkali-metal cluster Na_20_ (Solov’yov et al., 2002). Recently Asenjo et al. (Asenjo et al., 2013) showed that L-BFGS as well as FIRE algorithm is also a fastest minimizer and it led to less fragmented basins of attraction. As these magic number clusters are stable and have closed electronic and/or geometric shell, they can be used as building block in nanoscale materials and devices.

38, 75, and 98 atom Lennard-Jones clusters, truncated octahedron for LJ_38_ (Doye et al., 1999), tetrahedral symmetry for LJ_98_ (Leary and Doye, 1999a), Marks decahedra for LJ_75–77_ (Doye et al., 1995) and LJ_102–104_ (Doye and Wales, 1995) which are all multiple-funnel systems of the corresponding energy landscapes (Wales et al., 1998, Wales, 2004; Doye et al., 1999) were studied by GMIN optimization code by Wales Group (Wales, 2012; Oakley et al., 2013). Adaptive immune optimization algorithm (AIOA) search algorithm has been used for the structural optimization of monatomic LJ clusters (up to 200 atoms) (Ye et al., 2011; Cheng et al., 2004). Studies on the geometrical optimization of Lennard-Jones clusters within 250 atoms and Ag clusters (within 150 atoms) were carried out by adaptive immune optimization algorithm (AIOA) with dynamic lattice searching (DLS) operation (AIOA-DLS) using many-body Gupta potential (Wu and Wu, 2014a). Dynamic searching approach reduces the searching space and runs at a very high efficiency, especially for larger size clusters. This approach can be effectively used for other molecular or atomic clusters. The performance of DLS for the optimization of LJ clusters with 13 ≤ N ≤ 309 with different parameters are listed in Table 2. DLS method showed a very fast convergence speed compared with monotonic sequence basin-hopping (MSBH). Recently KLMC method is used to locate and explore double funnel landscape of LJ_38_ atom system (Lazauskas et al., 2017).

**TABLE 2:** Parameters Used in Dynamic Lattice Searching Method. For the magic numbers (38, 75–77, 98, 102–104). N_runs_ 10,000. (Shao et al., 2004b).

N	N_mov_	N_P_	N_try_	N_best_	N_runs_^a^
13–49	10	92	100	4	1,000
50–79	15	162	200	4	1,000
80–119	15	252	300	5	1,000
120–149	20	362	350	5	1,000
150–169	20	492	400	5	1,000
170–189	20	492	450	8	2000
190–199	20	492	500	10	2000
200–229	20	492	550	10	2000
230–251	20	642	550	10	2000
252–309	25	642	700	10	2000
500	40	812	1,000	10	10,000

**FIGURE 3:**
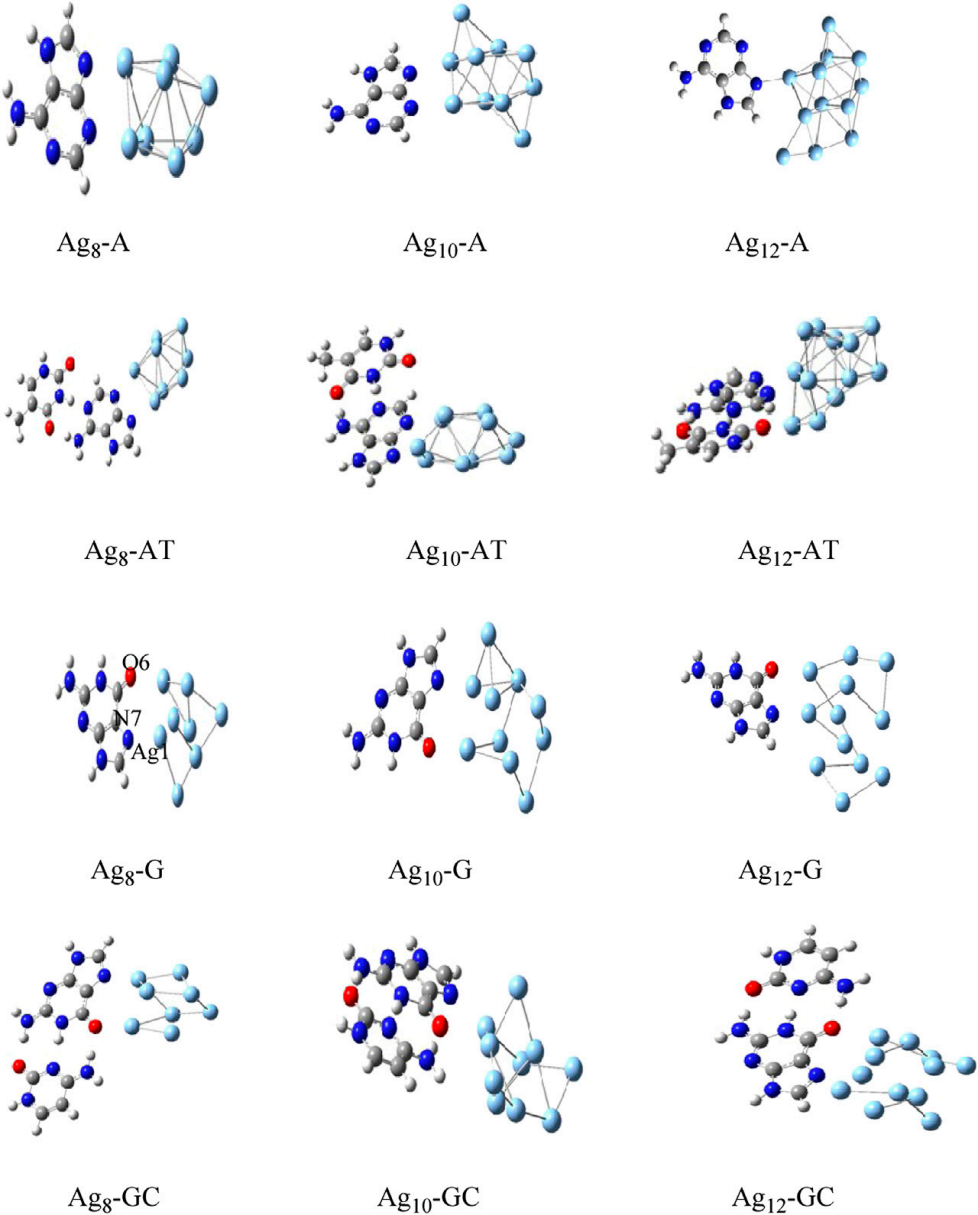
Optimized structures of Ag_n_-A,T/WC complexes for n = 8, 10 and 12 by DFT method (Srivastava, 2018a).

**FIGURE 4:**
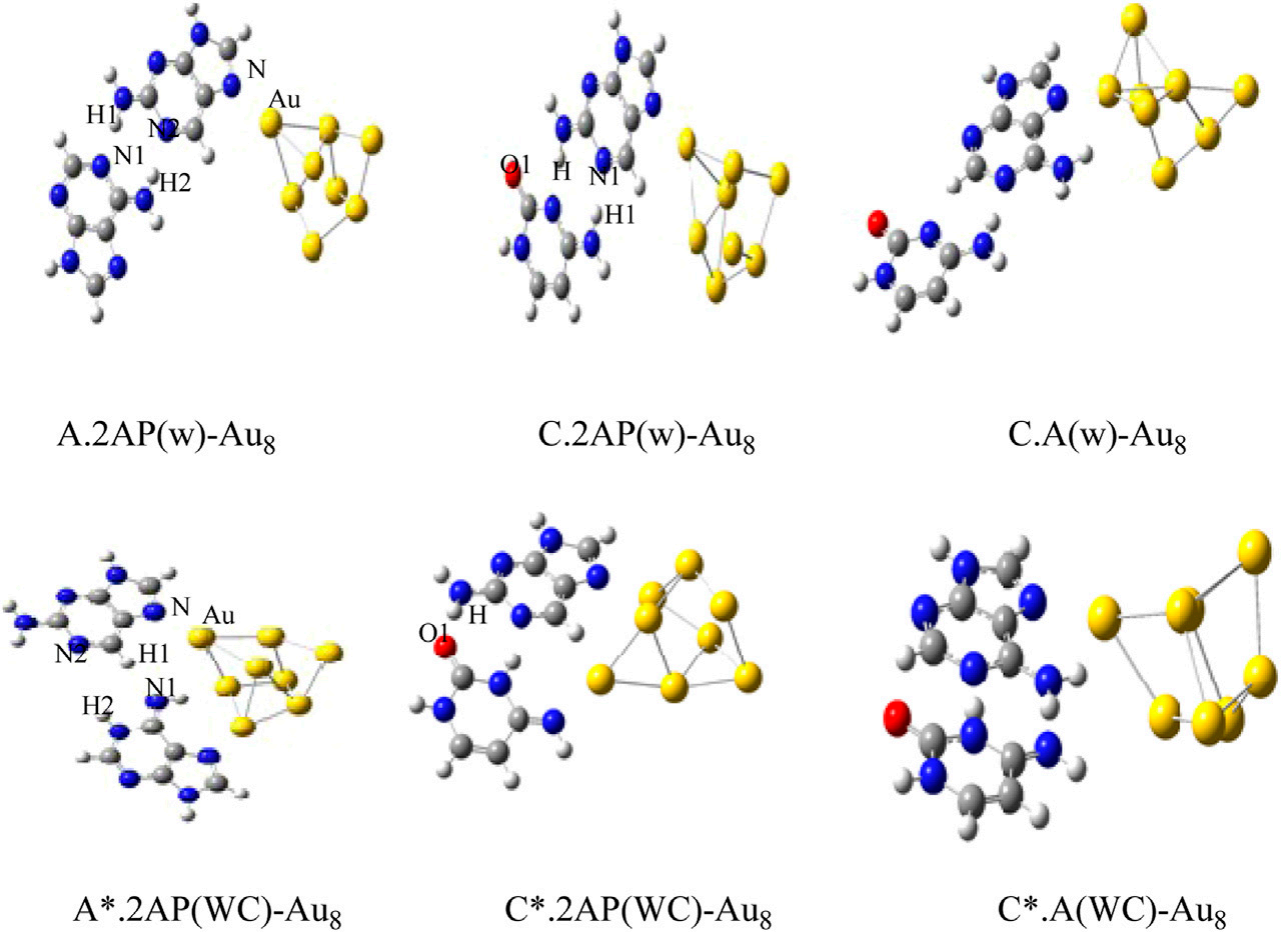
Optimized structures of Au_8_-wobble/WC mispair Complexes by DFT method. (Srivastava, 2018a).

The Gupta potential parameters with GMIN code were used for the gold and silver clusters for Cystene-coinage metal interactions (Srivastava, 2017a). The clusters were optimized using basin hopping algorithms within higher temperature and the structures validated the experimental studies. Results indicated that basin hopping algorithm based on the Monte Carlo minimization is appropriate for these clusters. The same GMIN code has been used for the structural optimization of silver clusters (Ag_8_, Ag_10_ and Ag_12_) in Ag_n_-A,T/WC complexes interactions (Srivastava, 2018a). See Figure 3. In Mutagen-Au_8_ complexes, the gold clusters were optimized using Gupta potential parameters and BH algorithm and the electronic and optical properties of the Mutagen-Au_8_ complexes (Srivastava 2017c) were studied with G09 software. See Figure 4.

### Bimetallic Clusters

In bimetallic clusters, four different types of chemical ordering are possible as core-shell, subcluster segregated (Janus nanoparticles), mixed and multiple shells. See Figure 1. The design of nanoalloys can be changed by fine tuning the mixing pattern. The chemical arrangement of these clusters are influenced by many factors as relative strengths of homo and heteronuclear bonds, atomic sizes, electronic/magnetic effects and many others (Ferrando et al., 2008; Oderji and Ding, 2011).

**FIGURE 5:**
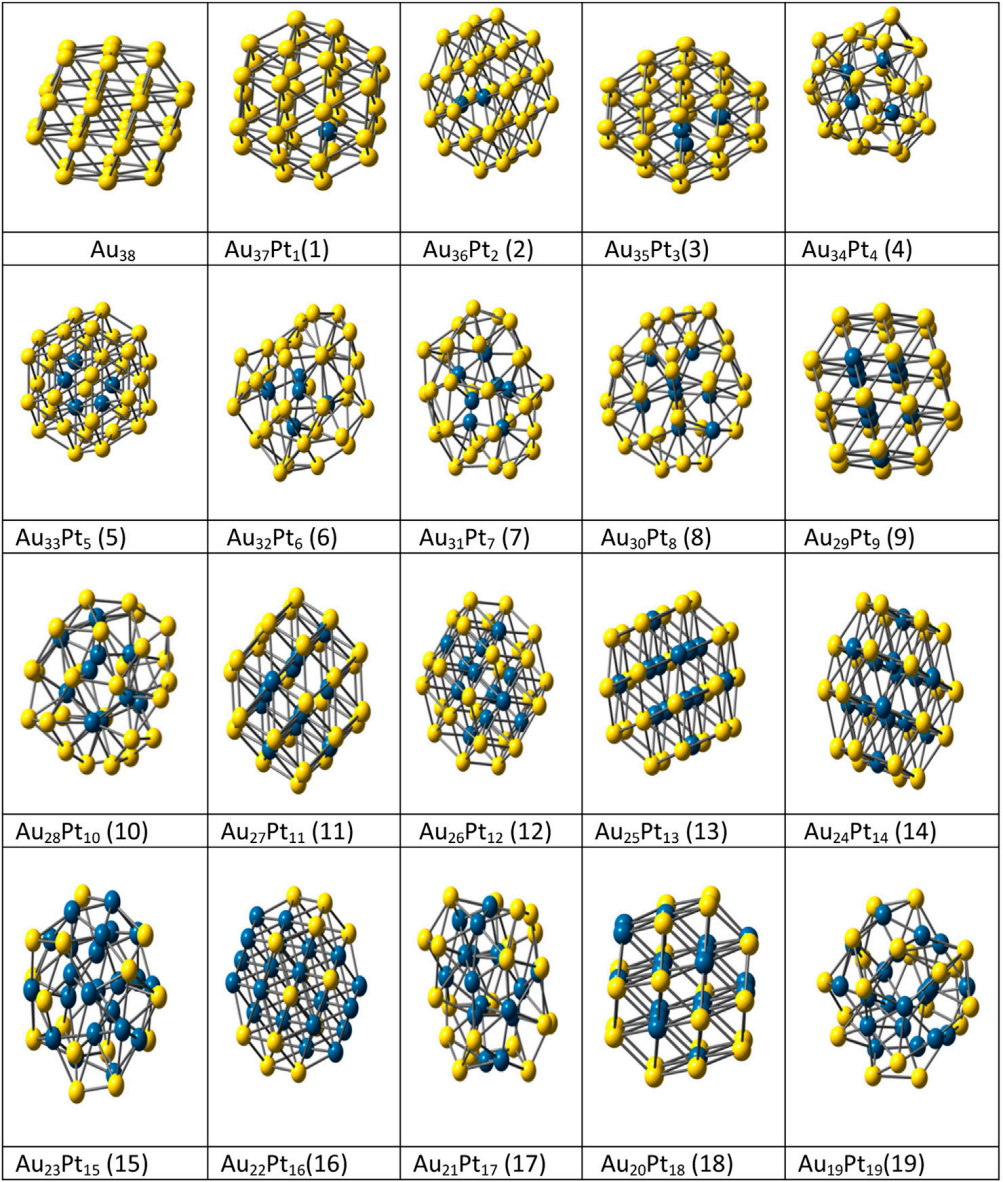
Optimized structures of Au_38-x_Pt _x_ bimetallic (x = 1–19) clusters. (Srivastava, 2018b).

The highly efficient unbiased optimization methods used for nanoalloys are the genetic algorithm (GA) (Daven et al., 1996; Xiang et al., 2004; Curley et al., 2007), basin hopping (BH) method and its variants (Wales and Doye, 1997; Wales et al., 1998; Leary and Doye, 1999b), self-consistent basin-to-deformed-basin mapping, heuristic algorithm (Leary and Doye, 1999b) with surface and interior operators (HA-SIO) (Takeuchi, 2006), fast annealing evolutionary algorithm (FAEA) (Cai and Shao, 2002), evolutionary algorithm (EA) (Hartke, 2000), random tunneling algorithm (RTA) (Shao et al., 2004a), dynamic lattice searching (DLS) methods (Pillardy et al., 1999; Shao et al., 2004b), modified adaptive immune optimization algorithm (AIOA) (Shao et al., 2004c) and Knowledge Led Master Code (KLMC) (Woodley, 2013) due to larger number of homotops. In a previous study Spin polarized density-functional theory (SP-DFT) (Pant and Rajagopal, 1972) with B3 (Becke, 1993) exchange functional and PW91 (Perdew and Wang, 1992) correlation functional was used to investigate small Ag_x_Pt_10-x_ (1 ≤ x ≤ 10) nanoalloys and global reactivity descriptors were used to determine the activity of these bimetallic clusters (Erlinda del et al., 2009). The LANL2DZ pseudo-potential with corresponding double- ζ basis set was used for Ag and Pt atoms (Hay and Wadt, 1985). Nanoalloys as iron and silver are of immense interest as they have distinct properties compared to the pure elemental clusters and corresponding bulk alloys due to finite size effects. These nanoalloys may show both magic sizes and magic compositions (Baletto and Ferrando, 2005). Paz Borbón et al. (Paz Borbón et al., 2008) used combined empirical potential (EP)/density functional (DF) method to study the structural properties and segregation effects for 38 atom binary clusters (combination of Pt-Ag, Ag-Au, Pd-Au and Ag-Pt) metals. Results favored mixed five-fold-symmetric/close-packed or decahedral arrangements for Pt-Pd, Ag-Pt and Ag-Au pairs. We have also used the combined EP-DF approach for structural optimization of nineteen bimetallic Au_38-x_Pt_x_ and Au_38-x_Ag_x_ clusters. The basin-hopping procedures with accept/reject strategies (10,000 Steps) were used and the studied structures were compared to the reference Au_38_ (theoretical and experimental) structure. Further the geometrical, thermal and other properties were studied for these binary structures (Srivastava, 2018b). See Figure 5.

A general tight-binding (TB) total energy scheme has been used to calculate the structural properties of the smaller bimetallic coinage metal compounds: Cu_3_Au, CuAu, CuAu_2_, CuAu_3_, and Cu_6_Au_8_ clusters (Metadjer et al., 2001). The structural and electronic properties of bimetallic gold-silver clusters (Bonacic et al., 2002) and Au_n_X_m_+ (X = Cu, Al, Y, In) (Bouwen et al., 1999) were studied by the TB energy scheme. *Heinebrodt el al.* (Heinebrodt el al., 1999) used DFT method for Au_n_X_m_ (X = Cu, Al, Y, In, Cs) clusters and found the electronic shell effects separately in the clusters. *Yuan et al.* investigated the geometric and electronic structures of Au_n_M (n = 1–7, M = Ni, Pd, Pt) by employing the first-principles method (Yuan et al., 2005). The structures and electronic properties of Cu_n_O_n_ (n = 1–8) clusters (Bae et al., 2011) were also carried out by DFT.

The structural stability and electronic properties of Pd_n_Si_q_ (n = 1–7 and q = 0, 1, −1) clusters were studied by DFT within GGA (Begum et al., 2014) framework. The chemical ordering in magic-size Ag–Pd clusters were carried out for global optimization searches with DFT based atomistic potential developed within the second-moment approximation to the tight-binding model (Cyrot-Lackmann and Ducastelle, 1971) using the BH algorithm (Wales and Doye, 1997; Rossi and Ferrando, 2009). BH algorithm consists of Metropolis Monte Carlo simulations in which local minimization are performed after each move. In this study exchange moves were only allowed to search the best homotops for the most favourable chemical ordering patterns. Thermodynamic parameters of surface configurations for the corresponding bulk alloys were studied by Strohl and King et al. (Strohl and King, 1989). In another study different chemical ordering patterns were analyzed for Ag–Pd nanoparticles up to 60 atoms (Bochicchio et al., 2014).

The embedded-atom method was used to study the structural stability of Cu_m_Ag_n_ nanoalloys with BH algorithm for all (m,n) with N = m + n from 2 to 60 atoms. Most of the structures were icosahedra, polyicosahedra, truncated octahedral and 5-fold pancakes (Molayem et al., 2010). The studies were compared to the Ni_m_Ag_n_ (Molayem et al., 2011) clusters using the similar approaches. Electronic shell closure effects have been observed for magic size N = 40 for Cu-Ag nanoalloys (Barcaro et al., 2006).

The second moment approximation to the tight binding (SMATB) method with Gupta potential and genetic algorithms (GA) were employed for the global optimization (Rossi et al., 2004; Barcaro et al., 2006; Nu_nez and Johnston, 2010) for CuAg clusters using three different algorithms; basin-hopping (BH) method, the energy-landscape paving method, and the parallel excitable walkers method. The predicted most stable clusters have core shell polyicosahedra structures (Rossi et al., 2004; Nu_nez and Johnston, 2010). Another study has been carried out for Au_19_X doped (X = Li, Na, K, Rb, Cs, Ag and Cu) clusters by *ab initio* scalar relativistic DFT method using ADF package based on zero-order regular approximation (ZORA) (vanLenthe et al., 1993; vanLenthe et al., 1994). Perdew-Wang 1991 (PW91) (Perdew, 1991) exchange-correlation (XC) functional within GGA with triple-STO basis set added with two polarization functions at the frozen core approximation level were used for the studies (Ghanty et al., 2010).

KLMC method (Woodley, 2013; Farrow et al., 2014) was used to study the nanocluster structures of binary heteropolar compounds; ZnO, MgO, KF, and CdSe, (Farrow et al., 2014) using interatomic potentials (IP) (within GULP (Gale, 1997; Gale and Rohl, 2003) and density functional theory (DFT) (within FHI-aims (Blum et al., 2009) respectively. The study showed that improved Lamarckian evolutionary algorithm within KLMC proves to be a useful tool for structural prediction for nanoclusters (Woodley, 2013). These algorithms were used for (BaO)_n_ nanoclusters (Escher et al., 2017) and it showed great resemblance for (KF)_n_ clusters, while (MgO)_n_ nanoclusters have barrel shape LM for n = 6. Interestingly (BaO)_n_ for n = (4, 6, 8, 10, 16) were considered to be the magic number clusters (Lazauskas et al., 2017). The similar code has been used to study the ionic semiconductor (ZnO)_1–32_ and CdSe (Farrow et al., 2014) to explore the energy landscape using interatomic potentials.

### Trimetallic and Tetrametallic Clusters

Trimetallic (or ternary) coinage metal clusters have shown potential applications in optics, electronics, magnetic, and catalytic field (Stucky et al., 1989; Teo et al., 1993; Hungria et al., 2006; Fang et al., 2011). These clusters are highly catalytic and selective, yet the studies are very complex (Toshima and Yonezawa, 1998). Various global optimization techniques are used for the structural analysis for these clusters. (See Introduction section paragraph 3).

The interactions of Cu-Ag-Au trimetallic clusters were described by the second-moment approximation of the tight-binding (TB) potentials (N-body Gupta potential) with AIOA method. AIOA is an adaptive heuristic GA based algorithm which is used in the biological applications also. Another modified algorithm, MAIOA is used for the structural optimization of bimetallic (Wu et al., 2009; Wu et al., 2017) and ternary clusters. The immune clone selection and a mutation operation were the basic steps of MAIOA algorithms (Wu et al., 2011). AIOA method was also approved for A_(l)_B_(m)_C_(n)_ (l + m + n = 9–55) clusters and A_(l)_B_(m)_C_(n_) (l = 13, m + n = 42) clusters (Wu et al., 2011). Further an improved adaptive immune optimization algorithm (AIOA-IC method) was found to be suitable for Cu_8_Au_n_Pt_30-n_ (n = 1–29), Cu_8_Au_n_Pt_47-n_ (n = 1–46), and partial 75, 79, 100, and 147 atom clusters (Wu et al., 2017). The structures, properties and interatomic interactions of trimetallic M–Pd–Pt (M = Ag and Au) upto 75 atoms were optimized using AIOA with the tight-binding Gupta potential (Wu et al., 2015). The Stacking fault (sf) and twin defects in Pd, Au–Pd, and Au–Pd–Pt clusters were studied with Gupta potential using DFT and fitted averaged parameters (Wu and Dong, 2014b). It was seen that the Gupta and Murrell Mottram potentials (Lloyd and Johnston, 2000) were found to be a good choice for the 19 atom trimetallic Double-icosahedra (DI) clusters (Farges et al., 1985). In another studies, random selection of bimetallic atom pairs were made with their exchanged location (Calvo and Yurtsever, 2004) based on the fact that the atoms with lower number of nearest neighbor contacts have higher potential energies in energy based mutations. Mutation and updating operation were performed for MAIOA runs till the achievement of maximal iteration number (LOOP), as the larger LOOP is needed for the structural optimization of unknown clusters. Limited memory quasi-Newton algorithm (L-BFGS) (Liu and Nocedal, 1989b) was used for local minimization (LM) for the clusters.

Au@Pd@Pt NPs were studied by DFT total-energy with VASP software (Zhou and Lee, 2007). The projector augmented wave method (PAW) and the Perdew–Burke–Ernzerhof generalized gradient approximation (GGA-PBE) was used for the exchange–correlation functional with an energy cut-off of 400 eV. A dipole correction (Neugebauer and Scheffler, 1992; Makov and Payne, 1995) was employed to use the induced dipole moment. Interestingly the theoretical results match well with the experimental values. In one of the Agpdpt alloys study, both empirical investigation and the theoretical analysis has given strong evidence that these nanoparticles are composed by an AgPd alloy core with Pt atoms lied on the surface (Wang and Yamauchi, 2011).

The structural optimization of (Au_m_-Ag_n_-Pd_o_-Pt_p_) (m = 10 and n + o + *p* = 10) tetrametallic coinage metal clusters (Srivastava, 2017b) were carried out using combined EP-DF method. Completely random starting configuration was taken subjected to the lowest DF energy minimizations within GMIN code. In this work, the thermal and chemical stability of thirty eight tetrametallic clusters were measured by various parameters. Mixing/segregation effect results indicated that the surface sites are occupied by Au and Ag atoms while inner and middle shell are occupied by Pt and Pd atoms. See Figure 2.

## Inorganic Fullerenes and Fullerene-Like Clusters

The stability, electronic and mechanical properties of fullerenes has been used broadly in nanotechnological and biomedical applications. The discovery of buckyball C_60_ (Kroto et al., 1985) has launched a new era and stimulated the search for other related allotropes of carbon as nanotubes, nanopeapods, nanocones, etc. Detailed information about the structure, synthesis, and properties of carbon fullerenes are published in books and reviews (Dresselhaus et al., 1996; Hirsch, 1999, 2002; Hirsch and Brettreich, 2005). In 1970s, the first articles about C_20_ and C_60_ cages and their boron derivatives using quantum-mechanical method was published by Bochvar et al. (Bochvar and Galpern, 1973; Bochvar and Galpern, 1974). There are other well-known fullerene-like and fullerenic allotropes, chalcogenides, halides and oxides (Bar-Sadan et al., 2007; Tenne and Seifert, 2009). Small cage-like clusters Si_n_ (n < 60) doped by endohedral metal atoms (Kumar, 2003) showed stable cage structures. The elemental fullerenes based on boron particles form pure boron and mixed borocarbide structures (Albert, 2009). In 1912 Stock reported his pioneering work on boranes (Stock and Massanez, 1912), which led to the identification of neutral boron hydrides (B_2_H_6_, B_4_H_10_, B_5_H_9_, B_5_H_11_, and B_6_H_10_). Further, Lipscomb and co-workers (Eberhardt et al., 1954) introduced the concept of three-center two-electron (3c-2e) bonding B_2_H_6_ diborane. The existence of regular octahedra of boron atoms in several metal hexaborides with general formula MB_6_ was demonstrated experimentally by Allard et al. (Allard, 1932; Pauling et al., 1934). Longuet-Higgins and Roberts show that the [B_6_]^2−^ unit has a highly stable closed-shell electronic arrangement (Longuet-Higgins and Roberts, 1954) and B_12_ icosahedron is a dominant structure of various allotropes of boron (Longuet-Higgins and Roberts, 1955). The existence of other deltahedral boranes, B_11_H_11_^2−^, B_9_H_9_^2−^, B_8_H_8_^2−^, B_7_H_7_^2−^, and B_6_H_6_^2−^ were demonstrated in experimental studies (Klanberg and Muetterties, 1966; Klanberg et al., 1967; Boone, 1964). In another study to search global minima for the B_*n*_H_*n*__+2_ (*n* = 2–5) series, it was found that classical structures composed of 2c-2e B—H and B—B bonds become progressively less stable. The reason for this might be that the boron atoms are trying to avoid sp^2^ hybridization and trigonal structure at the boron atoms, which is highly unfavourable as in that case one 2p-AO is empty (Osorio et al., 2012). These studies play a very important role to emulate structures analogous to the C allotropes (i.e., C_60_), such as in systems containing N-B replacing an isoelectronic C-C fragment. A lot more about boron cluster studies is covered in an extensive review by Alexandrova et al. (Alexandrova et al., 2006). The most stable allotropes of boron exists as B_12_ (boron polyhedral) while sandwich-like hexagonal-structured “metal” atoms between boron atomic structure is seen in layered borides MgB_2_, AlB_2_, and TiB_2_ (Chernozatonskii, 2001; Ivanovskaya et al., 2004). The most stable crystalline form of binary boron nitride is the hexagonal one, which is held by weak van der Waals interaction. Semi-empirical Hückel Austin Model (AM1) and *ab initio* Hartree–Fock (HF) methods have predicted possible configurations for BN nanoalloys. Similar approach with coupled-cluster theory (CCSD) method showed stable cage structures for (BN)_n_ (n = 8–11) clusters. DFT tight-binding method (DFTB) was performed to study the structural stability of (BN)_n_ fullerenes with n = 4–30 (Fowler et al., 1996) and (BN)_n_ fullerenes with n = 13–35 (Rogers et al., 2000). Octahedron-like fullerenes structures were found for B_12_N_12_, B_16_N_16_, and B_28_N_28_ (Seifert et al., 1997). *Ab initio* calculations have been performed for energetically stable small cages B_13_N_13_, B_14_N_14_, B_16_N_16_ clusters (Strout, 2004) and B_n_N_m_ fullerenes for 20 < (n + m) < 288 (Alexandre et al., 2001; Batista et al., 2006). Semi-empirical PM5 and discrete-variational calculations were carried out for B_12_N_12_, B_28_N_28_, B_36_N_36_ (Oku et al., 2004a; Oku et al., 2004b; Oku et al., 2004c) and B_24_N_24_ (Oku et al., 2003) clusters, while semi-empirical AM1 and discrete-variational (DV) calculations were used to study the stability of single atom doped B_36_N_36_ fullerene clusters (Nishiwaki et al., 2004). First principle calculations were carried out for boron-carbon nanocages (B_12_C_48_, B_12_C_50_) and it was found that these structural motifs showed aggregated boron atoms at one location in the form of a patch. These studies have violated all the other previous suggested empirical rules for constructing low-energy fullerenes. Also the energetic stabilities of these two clusters predicted that the structures derived from the C_60_ (buckminsterfullerene) are not necessarily magic sizes for heterofullerene structures (Mohr et al., 2014).

*Ab initio* projector-augmented wave (PAW) spin-polarized calculations were performed on La@B36N36 endofullerene, tetrahedral Fe_4_, Co_4_, and Ni_4_ clusters encapsulated into B_36_N_36_ fullerene (Nigam and Majumder, 2007), while spin polarized DFT–GGA pseudo-potential calculations were performed for B_36_N_36_ fullerene doped by (Fe, Co, and W) and FeO molecule (Batista et al., 2007). The stability of BN tubular structures were studied by DFT calculations, while the sphericity of BN cap models (squares, and pentagon–pentagon and pentagon–heptagon pairs) (Fowler et al., 1999) and octahedral fullerenes B_12_N_12_ inside (14,0), (8,8), and (12,4) BN nanotubes (Enyashin and Ivanovskii, 2008) were studied by DFTB method. Different lattice parameters of B_12_N_12_ and B_24_N_24_ (Pokropivny and Bekenev, 2006, 2007) have been studied by semi-empirical MNDO and *ab initio* FLAPW methods (Pokropivny et al., 2000) and the results indicated that B_12_N_12_ fullerites were the most stable diamond-like lattice structure.

In a latest study entirely unusual derivatives of boron clusters doped with lithium, LiB_*n*_^0/−^ (*n* = 10–20) clusters were studied through Crystal structure AnaLYsis by Particle Swarm Optimization (CALYPSO) structural search approach alongwith the DFT calculations. Three (half-sandwich-type, quasi-planar and drum-type) structures were found for the studied clusters (Shi et al., 2020). The lowest-energy minima of the pure B_22_ cluster and the capacity of its isomers to form endohedrally doped cages with two transition metal atoms M (M = Sc and Ti) were carried out with genetic search algorithm using DFT calculations (Celaya et al., 2020). Recent reviews on boron clusters (Yan et al., 2020; Axtell et al., 2018; Zhu and Hosmane, 2018; Núñez et al., 2016a; Núñez et al., 2016b) are recommended for the interested readers.

DFTB method was used to investigate the properties of sulfide fullerene-like particles and sulfide MoS_2_ nanotubes, while DFTB calculations with a derived continuum approach (Bar-Sadan et al., 2006.; Enyashin et al., 2007) was used to investigate WS_2_, MoS_2_, and MoSe_2_ polyhedral nanoclusters (Margulis et al., 1996; Parilla et al., 2004). The semi-empirical Extended Hückel Theory (EHT) level was used to study (MoS_2_)_n_ upto (n = 64), (MoS_2_)_n_ upto (n = 576), imperfect MoS_2_ fullerenes and nanoseashells (Enyashin et al., 2009). Halide fullerenes was studied by *ab initio* spin-polarized DV method without geometry optimization for (NiCl_2_)_48_, (FeCl_2_)_48_, and (CdCl_2_)_48_ clusters (Enyashin and Ivanovskii, 2005a; Enyashin et al., 2005b), while (TiO_2_)_n_ nanooctahedra with n upto 108 (Enyashin and Seifert, 2007) were studied by DFTB method. Recently KLMC is used to investigate the metallic Ni_13_ and covalently bonded C_60_ (buckminsterfullerene) potential energy surfaces (Lazauskas et al., 2017).

P, As, Sb, and Bi, are the few elements among all the variety of periodic table which can appear as hexagonal atomic layers assembled using covalently bounded sp^3^ hybridized atoms. The possibility of stable non-carbon fullerenes as phosphorus fullerene-like cage structures was studied on the basis of Density Functional Tight Binding calculations (Seifert et al., 2001). DFT calculations showed that P_20_ (dodecahedron) is the most stable structure and P_n_ hollow cages corresponds to the metastable structures. It was observed that with increasing nuclearity these metastable structures become less stable with respect to separate molecular P_4_ units.

## Dipolar Clusters

Dipolar interactions form chains with a low coordination number, while spherical particle clusters with isotropic attraction form close-packed structures. The dipolar clusters favor strongly distinguished nearest-neighbour interactions. The combination of both isotropic and dipolar interactions form intricate knot, link and coil structures. The global minima of these interconverted self-organize structures are bound by the Stockmayer potential (Lennard-Jones plus point dipole). The Stockmayer model with dipolar fluids has been summarized in a good review (Teixeira et al., 2000). For these particles, the energy landscape for low-lying minima was obtained by the basin-hopping global optimization. The isotropic Lennard-Jones part of the potential is used to drive the compact structures toward highly-coordinated arrangements for the frustrated Stockmayer clusters, while chain-like motifs are favored by dipolar interactions. The studies of these idealized model systems are useful as it give insight for the knot systems as biomolecules and synthetic organic molecules.

The global optimization calculations for the Stockmayer particle have been carried out for the energetically favorable structures (knots, links, and coils) with a permanent dipole plus anisotropic soft core and alluring tail by Miller et al. (Miller and Wales, 2005). These particles have anisotropic point dipole with five degrees of freedom and cylindrical symmetry. Further, the PES is characterized by scaling out local minima which corresponds to the locally stable structures, connected *via* first-order saddle points (transition states). The clusters of spherical particles are bound by Lennard-Jones and Morse type of simple isotropic potentials, while the anisotropic Stockmayer potential differs from these potentials as the particles' have the tendency to form chains (Farrell et al., 2013).

## Energy Landscape for Kagome Lattice from Soft Anisotropic Particles

Studies were carried out for the energetically stabilized kagome structures, which are the simple model of triblock Janus particles based on the discoidal building blocks. Basin-hopping global optimization was used for these particles. The three nearest neighbors were detected by an algorithm based on the interparticle distances. Further it was seen that the energetic stabilization is enhanced with the occurrence of sedimentation. The interaction of each ellipsoid of two building blocks occured via the Paramonov–Yaliraki (PY) potential (Paramonov and Yaliraki, 2005). The Paramonov-Yaliraki (PY) potential is used to study the collection of both homomolecular and heteromolecular pyrene, coronene, and circumcoronene below 1000 K within a stochastic Monte Carlo framework (Hernandez-Rojas and Calvo, 2019). This elliptic potential is also applicable for mixtures of any (size, orientation) ellipsoids and/or spheres, hard and soft particles.

## Energy Landscape for Planar Colloidal Systems

Short-ranged pairwise Morse potential is more appropriate method to study the structural optimization of colloidal clusters with planar morphologies. The PES, global minima, rearrangement paths with discrete path sampling and free energy landscapes are visualized by the disconnectivity graphs. Here the number of nearest neighbor contacts control the short range potential. It was found that the free energy global minimum differs from the potential energy GM in quasi-degeneracy state due to the symmetry effects, which results in higher entropic lower symmetry structures. BH steps were taken as random Cartesian displacements (Wales, 2004) and the possible nominee for the transition states are selected between the minima’s by the doubly-nudged (Trygubenko and Wales, 2004; Carr et al., 2005) elastic band (Henkelman et al., 2000a; Henkelman and Jónsson, 2000b; Henkelman and Jónsson, 2001) method.

## Energy Landscapes for Water Dimer

The understanding of the structure and thermodynamics of water is very important as water is used in the wide range of applications from biomolecular solvation to the atmospheric chemistry. The angle axis framework with TIP4P potential was used for the water clusters containing eight molecules. In these clusters the energy landscape was mapped with the basin-hopping global optimization and a modified limited-memory Broyden-Fletcher-Goldfarb Shanno (L-BFGS) algorithm to find the global minima and a database for the low energy minima. The combined doubly-nudged elastic band and a hybrid eigenvector-following method (Henkelman and Jónsson, 2000b; Kumeda et al., 2001.) was used to obtain minimum-transition state-minimum triplets. The intermolecular and intramolecular forces were studied over the larger amplitudes to get a connectivity PES graph by exploring the local minima. Recently artificial bee colony (ABC) algorithm with “ABCluster” was used to find the successful location of global minima for TIP4P water clusters (H2O)_N_ (N ≤ 20). The similar methodology was further applied to various clusters of different chemical nature: 10 microhydration clusters, 4 methanol microsolvation clusters, 4 nonpolar clusters and 2 ion–aromatic clusters (Zhang and Dolg, 2016).

## Energy Landscapes of Hydrated Sulfate Clusters

These clusters were optimized using BHMC simulations with a rigid-body EP and a move set, which included the cycle inversions to inquire the hydrogen bond topologies with the sulfate ion, belonging to the Hofmeister series. As the system has large size so the bond parameters (length, angle) of the sulfate clusters were held rigid. The water molecules were described by four-site rigid-body TIP4P water potential (Jorgensen et al., 1983a; Jorgensen et al., 1983b; Jorgensen et al., 1983c; Kazachenko and Thakkar, 2010) to describe the water phase diagram with certain modifications. The TS connecting the minima on the PES were located by doubly nudged elastic band method. Translational (linear interpolation) and rotational coordinates (spherical interpolation) were used for the endpoints interpolation and the artificial frustration was removed by connecting the minima to the global minimum (Małolepsza et al., 2010) with the visualization of disconnectivity graphs (Becker and Karplus, 1997; Morgan and Wales, 2014; Smeeton et al., 2014).

## Conclusion

The rise of machine learning (ML) has explored the use of these algorithms in atomistic modeling and inference techniques and led it toward the data-driven approaches in the recent years. The machine learning landscape can easily analyze the most fitted functions that exhibit multiple solutions as local minima. Supervised as well as the unsupervised learning methods in combination to the fundamental mathematical concepts are mostly used for the machine learning techniques (Ceriott, 2019). These studies are mainly focused toward clusters, biomaterials, crystals and self-organized structures (Ballard et al., 2017). ML can use the algorithms more effectively to get a new and useful insight about the corresponding predictions, so that the directions for new interdisciplinary research will be explored. Though certain limitations regarding the use of machine-learning techniques in atomistic modeling are still needed to be rectified for materials, chemical, and biomolecular clusters, we hope that machine learning techniques and computational chemical physics will collaborate in highly efficient manner to show great productivity for different models in near future.
